# Accelerated partial breast irradiation using multicatheter brachytherapy for select early-stage breast cancer: local control and toxicity

**DOI:** 10.1186/1748-717X-5-56

**Published:** 2010-06-19

**Authors:** Seung-Gu Yeo, Juree Kim, Geum-Hee Kwak, Ji-Young Kim, Kyeongmee Park, Eun Seok Kim, Sehwan Han

**Affiliations:** 1 Department of Radiation Oncology, Soonchunhyang University College of Medicine, Cheonan, Korea; 2 Department of Radiation Oncology, Inje University Sanggye Paik Hospital, Seoul, Korea; 3 Department of Surgery, Inje University Sanggye Paik Hospital, Seoul, Korea; 4 Department of Radiology, Inje University Sanggye Paik Hospital, Seoul, Korea; 5 Department of Pathology, Inje University Sanggye Paik Hospital, Seoul, Korea; 6 Department of Radiation Oncology, Kwandong University Jeil Hospital, Seoul, Korea

## Abstract

**Background:**

To investigate the efficacy and safety of accelerated partial breast irradiation (APBI) via high-dose-rate (HDR) multicatheter interstitial brachytherapy for early-stage breast cancer.

**Methods:**

Between 2002 and 2006, 48 prospectively selected patients with early-stage breast cancer received APBI using multicatheter brachytherapy following breast-conserving surgery. Their median age was 52 years (range 36-78). A median of 34 Gy (range 30-34) in 10 fractions given twice daily within 5 days was delivered to the tumor bed plus a 1-2 cm margin. Most (92%) patients received adjuvant systemic treatments. The median follow-up was 53 months (range 36-95). Actuarial local control rate was estimated from surgery using Kaplan-Meier method.

**Results:**

Local recurrence occurred in two patients. Both were true recurrence/marginal miss and developed in patients with close (< 0.2 cm) surgical margin after 33 and 40 months. The 5-year actuarial local recurrence rate was 4.6%. No regional or distant relapse and death has occurred to date. Late Grade 1 or 2 late skin and subcutaneous toxicity was seen in 11 (22.9%) and 26 (54.2%) patients, respectively. The volumes receiving 100% and 150% of the prescribed dose were significantly higher in the patients with late subcutaneous toxicity (p = 0.018 and 0.034, respectively). Cosmesis was excellent to good in 89.6%.

**Conclusions:**

APBI using HDR multicatheter brachytherapy yielded local control, toxicity, and cosmesis comparable to those of conventional whole breast irradiation for select early-stage breast cancer. Patients with close resection margins may be ineligible for APBI.

## Background

Over the last decades, breast-conserving surgery (BCS) followed by whole breast irradiation (WBI) became the standard of care for the treatment of early-stage breast cancer. However, the 5-6 weeks of conventional WBI are problematic for elderly patients, working women, and those who live a great distance from a radiotherapy facility [1]. In addition, controversies and logistical problems exist that are associated with integrating this prolonged course of WBI and systemic chemotherapy [2]. These make a barrier to the acceptance of breast conservation by patients or their physicians, and some patients do not receive WBI after BCS [3].

The rationale underlying the accelerated partial breast irradiation (APBI) is that in-breast failure for select low-risk patients occurs mostly in the immediate area of the tumor bed [4]. The risk of failing at a location remote from the tumor bed is very low; it occurs despite WBI and its rate is similar to that of new contralateral breast cancer [5,6]. Accordingly, standard elective irradiation of the entire breast for presumed occult disease can be replaced with partial breast irradiation. The reduction in irradiation volume allows the administration of a larger fraction dose in a shorter period without significant additional toxicity. In addition, WBI-induced cardiovascular mortality, which counteracted an increase in overall survival by adding WBI after BCS, may be reduced using APBI by avoiding radiation exposure to coronary vessels [7].

Several centers have evaluated the feasibility and efficacy of APBI and produced evidences supporting the use of APBI for select early-stage breast cancer patients [8-10]. We pioneered APBI in our country, and this is the first report of long-term outcomes. The study investigated the efficacy and safety of APBI using high-dose-rate (HDR) multicatheter interstitial brachytherapy for select early-stage breast cancer patients and the endpoints were the local control rate, treatment toxicity, and cosmesis.

## Methods

### Patients

This study included 48 prospectively selected women with breast cancer in whom adjuvant radiotherapy was performed using interstitial brachytherapy alone after BCS. They were treated between May 2002 and December 2006, at the Inje University Sanggye Paik Hospital. We recommended interstitial brachytherapy for patients who met all of the following criteria: (1) age > 35 years, (2) tumor size ≤ 4 cm, (3) negative surgical margin, (4) negative axillary lymph nodes or singular positive node without extracapsular extension, (5) suitable breast anatomy for implantation, and (6) full recognition of possible increased risk of local failure. Patients with invasive lobular histology, extensive intraductal carcinoma, or multifocality were excluded. The study was approved by our institutional review board and written informed consent was obtained from the patients.

The median patient age was 52 years (range 36-78). Histological subtypes were invasive ductal carcinoma in 36 (75.0%) patients, medullary carcinoma in 6 (12.5%), ductal carcinoma *in situ *in 5 (10.4%), and tubular carcinoma in 1 (2.1%). The pathological T classification was Tis in 5 (10.4%), T1 in 28 (58.3%), and T2 in 15 (31.3%). The pathological N classification was N0 in 44 (91.7%) and N1 in 4 (8.3%); one positive node without extracapsular extension was found out of 6-16 nodes retrieved. The pathological resection margin was clear (≥ 0.2 cm) in 42 (87.5%) and close (> 0, < 0.2 cm) in 6 (12.5%) patients. Further information on the patient, tumor, and treatment characteristics is given in Table 1.

**Table 1:** Patient, tumor, and treatment characteristics

		No (%)
Age		
	Range	36-78
	Median	52
Tumor size (cm)		
	Range	0.4 - 4.0
	Median	1.5
T classification		
	Tis	5 (10.4)
	T1	28 (58.3)
	T2	15 (31.3)
N classification		
	N0	44 (91.7)
	N1	4 (8.3)
Histological subtype		
	Invasive ductal	36 (75.0)
	Invasive medullary	6 (12.5)
	Ductal carcinoma *in situ*	5 (10.4)
	Invasive tubular	1 (2.1)
Hormone receptor		
	ER + and PR +	34 (70.8)
	ER + and PR -	3 (6.3)
	ER - and PR -	11 (22.9)
Resection margin		
	Clear (≥ 0.2 cm)	42 (87.5)
	Close (> 0, < 0.2 cm)	6 (12.5)
Radiation therapy		
	34 Gy (3.4 Gy/fraction)	40 (83.3)
	30 Gy (3.0 Gy/fraction)	8 (16.7)
Systemic therapy		
	Chemotherapy + Hormonal therapy	24 (50.0)
	Hormonal therapy	15 (31.3)
	Chemotherapy	5 (10.4)
	None	4 (8.3)

*Abbreviation: *ER = estrogen receptor; PR = progesterone receptor.

### Treatments

All patients underwent BCS with gross total resection of the primary tumor and a sentinel node biopsy (n = 29, 60.4%) or level I/II axillary dissection (n = 19, 39.6%). Four titanium clips were positioned at the excision cavity boundaries: superiorly, inferiorly, medially and laterally. Four to six (median 5) guide needles were inserted during surgery. A single (n = 17, 35.4%) or double (n = 31, 64.6%) plane implant was performed. The needles were separated from each other by 1-1.5 cm. The distance from the implant plane to the thoracic wall or overlying skin should not be < 1 cm. The needles were replaced with flexible catheters and fixed with buttons.

Radiotherapy was started after receiving complete histological reports, at an interval of 6-9 days after surgery. The radiotherapy was planned using the PLATO brachytherapy planning system (Nucletron BV, Veenendaal, The Netherlands). Two post-implant isocentric radiographs were taken on a simulator with variable angles and used for digitizing and three-dimensional reconstruction of the catheters and clips. The dose points were related to the active source positions, and they were placed at a given distance (0.5-1 cm) from the catheters. The distance and active source positions were defined individually for each catheter, considering the location of the clips. The size of the planning target volume was estimated in such a way that the reference dose points were 1-2 cm from the clips in each direction. Then, the dose points and geometry were optimized [11]. The median prescribed reference dose was 34 Gy (n = 40) in 10 fractions bid separated by a minimum 6-h interval within 5 days. Eight patients received 30 Gy in 10 fractions bid. Patients were treated in the supine position using the microSelectron HDR remote afterloading equipment with iridium-192 (Nucletron BV). Before each radiotherapy session, a radiation oncologist monitored the patients for complications and checked the catheter placement. If not all of the pathological criteria for sole interstitial brachytherapy were met, then the interstitial brachytherapy was converted to boost irradiation followed by a course of WBI and these patients were excluded from this study.

To estimate the skin dose, a flexible wire cross was positioned on the skin surface as representatively as possible above the active source positions. During the process of digitizing the implants, the dose points were also assessed with the help of two isocentric radiographs. Representative skin point doses were calculated and the maximum skin dose was documented for each patient.

Chemotherapy was given to 29 (60.4%) patients starting within 9 days of the start of brachytherapy: cyclophosphamide, methotrexate, and 5-fluorouracil in 26 and doxorubicin, cyclophosphamide, and docetaxel in 3. Thirty-nine (81.3%) patients received hormonal therapy: tamoxifen in 22 and an aromatase inhibitor in 17.

The patients were seen every 3 months for the first 2 years and every 6 months thereafter, with a physical examination, chest X-ray, and blood tests. Mammography and ultrasound examinations of the breast and abdomen were performed at 6 months after APBI and then yearly thereafter.

### Analysis

To quantify the dose distributions, volume parameters and the dose homogeneity index (DHI) were calculated using dose-volume histograms. The volume parameters included the volumes receiving 100% and 150% of the prescribed dose (V_100 _and V_150, _respectively). The DHI was calculated as (V_100 _- V_150_)/V_100_, and was used to assess the dosimetric quality.

Local recurrence was defined as the recurrence of cancer in the treated breast proven histologically. A true recurrence/marginal miss was defined as a recurrence within or immediately adjacent to the primary tumor site. An elsewhere recurrence was defined as a local recurrence detected at least 2 cm from the surgical clips [12]. The actuarial rate of local recurrence was estimated from the date of surgery using the Kaplan-Meier method.

The cosmetic evaluation was based on the standards set forth in the Harvard criteria, which consisted of a four-tiered grading system: excellent, good, fair, and poor [13]. Late toxicity of the skin and subcutaneous tissue was scored according to the Radiation Therapy Oncology Group (RTOG)/European Organization for Research and Treatment of Cancer late radiation morbidity scoring scheme [14]. The cosmesis and toxicity scores recorded at the last follow-up were analyzed. To analyze the association between the dosimetric parameters or chemotherapy use and treatment toxicity, *t*-test, Fisher's exact test, or the chi-square test was used, as appropriate. A p-value of < 0.05 was deemed statistically significant. All statistical tests were performed using SPSS software (release 14.0; SPSS Inc., Chicago, IL, USA).

## Results

### Local control

The median follow-up period was 53 months (range 36-95) and there was no death. Local recurrence occurred in two patients 33 and 40 months after surgery. Both were true recurrence/marginal miss and developed in patients with close surgical margin (Table 2). The 5-year actuarial local recurrence rate was 4.6%. No regional nodal or distant metastasis was detected. The patients with recurrences received salvage surgery and all patients were alive without evidence of disease at the last follow-up.

**Table 2:** Characteristic of the patients with local recurrence

No	Age	Pathology	Tumor size (cm)	pN	ER	PR	RM	Radiation therapy	Systemic therapy	Failure	Time to failure (mo)	Salvage surgery	Follow-up after salvage surgery (mo)
1	52	DCIS	1.8	0	+	-	Close	34 Gy	TAM	TR/MM	33	BCS	21
2	42	IDC	2.2	0	+	+	Close	34 Gy	CMF/TAM	TR/MM	40	MRM	13

*Abbreviation: *pN = pathological nodal classification; ER = estrogen receptor; PR = progesterone receptor; RM = resection margin; DCIS = ductal carcinoma *in situ*; IDC = invasive ductal carcinoma; TAM = tamoxifen; CMF = cyclophosphamide, methotrexate, 5-fluorouracil; TR/MM = true recurrence/marginal miss; BCS = breast-conserving surgery; MRM = modified radical mastectomy.

### Dosimetry, toxicity, and cosmesis

The mean V_100 _and V_150 _values were 44.7 ± 17.9 cm^3 ^(range 12-101) and 22.8 ± 8.3 cm^3 ^(range 5-46), respectively. The mean DHI was 0.5 ± 0.03 (range 0.44-0.57). The maximum skin dose ranged from 12-69% (median 39%) of the prescribed dose.

Early side effects were usually mild and the breast pain, edema, or erythema subsided with conservative management. Grade 1 and 2 late skin toxicity occurred in 8 (16.7%) and 3 (6.3%) patients, respectively. Grade 1 and 2 late subcutaneous toxicity developed in 19 (39.6%) and 7 (14.6%) patients, respectively. Asymptomatic fat necrosis was detected on routine follow-up mammography in 5 (10.4%) patients, but required no surgical intervention. Dosimetric parameters like the V_100_, V_150, _and DHI did not differ significantly according to the occurrence of late skin toxicity. V_100 _and V_150 _were significantly higher in the patients with late subcutaneous toxicity (p = 0.018 and 0.034, respectively) (Table 3). The maximum skin dose was 43 ± 12% and 37 ± 11% in the patients with and without late skin toxicity, respectively (p = 0.190). The rates of late treatment toxicities did not differ according to the use of chemotherapy. Cosmesis was excellent (n = 34) or good (n = 9) in 89.6% of the patients. No one had poor cosmesis.

**Table 3:** Comparison of dosimetric parameters according to the late treatment toxicity

Toxicity		No (%)	V_100 _(cm^3^)	*P*^‡^	V_150 _(cm^3^)	*P*^‡^	DHI	*P*^‡^
Skin								
	Yes*	11 (22.9)	51.3 ± 12.1	0.221	25.7 ± 6.2	0.222	0.51 ± 0.03	0.105
	No	37 (77.1)	43.0 ± 18.9		22.0 ± 8.7		0.49 ± 0.03	
Subcutaneous tissue								
	Yes^†^	26 (54.2)	50.2 ± 18.2	0.018	25.1 ± 8.1	0.034	0.50 ± 0.03	0.099
	No	22 (45.8)	37.5 ± 15.0		19.9 ± 7.8		0.48 ± 0.03	
Fat necrosis								
	Yes	5 (10.4)	40.4 ± 18.7	0.529	21.0 ± 9.8	0.560	0.48 ± 0.02	0.465
	No	43 (89.6)	45.4 ± 17.9		23.1 ± 8.1		0.49 ± 0.03	

*Abbreviation: *V_100 _and V_150_= volumes receiving 100% and 150% of the prescribed dose; DHI = dose homogeneity index, (V_100 _- V_150_)/V_100_.

*Grade 1 to 2 toxicity.

^†^Grade 1 to 2 toxicity. Five patients with asymptomatic fat necrosis also had grade 2 subcutaneous toxicity.

^‡^*t*-test.

## Discussion

Long-term results of the phase II multicenter APBI trials for select early-stage breast cancer were recently reported [8-10,15]. In the RTOG phase II trial [9], 66 patients received HDR brachytherapy (34 Gy in 3.4 Gy bid for 5 days) and 33 patients received low-dose-rate brachytherapy (45 Gy). The estimated 5-year local recurrence rate was 4% (3% in HDR and 6% in low-dose-rate) after a median follow-up of 7 years. In the German-Austrian phase II trial [10], 175 patients received pulsed-dose-rate brachytherapy (49.8 Gy) and 99 received HDR brachytherapy (32 Gy in 4 Gy bid for 4 days). After a median follow-up of 32 months, the 3-year local recurrence rate was 0.4%. In the single-institution phase II trial conducted in Hungary [8], 45 patients received HDR brachytherapy, either 36.4 Gy (n = 37) or 30.3 Gy (n = 8) in seven fractions for 4 days. After a median follow-up of 81 months, the 5-year local recurrence rate was 4.4%, which was not significantly different from that for WBI in a retrospective comparative analysis. Overall, recently published APBI studies using multicatheter interstitial brachytherapy reported annual local recurrence rates below 1%, which is equivalent to the outcomes of WBI [8,16]. We found a 4.6% 5-year local recurrence rate, which translates to an annual recurrence rate of 0.9%, and is not different from those of other institutions.

Patients who have a substantial chance of harboring residual disease located a significant distance from the edge of the excision cavity or who potentially have multicentric disease have been precluded from APBI trials. The eligibility criteria of the RTOG trial included unicentricity, T1 or T2 (≤ 3 cm), infiltrating nonlobular carcinoma, pathologically negative margin, N0 or N1 without extracapsular extension, and no extensive intraductal component [9]. The eligibility criteria of the German-Austrian trial included tumor diameter ≤ 3 cm, clear resection margin (≥ 0.2 cm), N0 or singular nodal micrometastasis, estrogen and/or progesterone receptor positive, and ≥ 35 years [10]; patients were excluded if multifocality, poor differentiation, an extensive intraductal component, or lymphovascular invasion existed. Regarding resection margin, the American Brachytherapy Society recommended a negative margin, whereas the American Society of Breast Surgeons recommended a margin of at least 0.2 cm [4]. We experienced two local recurrences which were true recurrence/marginal miss and occurred to the patients with close resection margin. Positive margin status is generally accepted as a major risk factor for local recurrence after BCS and radiotherapy, and the width of clear surgical margins significantly influences local tumor control [17]. At least 0.2 cm tumor-free margins are deemed acceptable in some APBI trials [10,18], but others also successfully treated patients with close margins by APBI [8,9]. However, recently published recommendations for APBI selection criteria categorized patients with close margins as an intermediate-risk group, as there are only limited data supporting the use of APBI for these patients [19]. Our results may indicate that close margins should be an exclusion criterion for APBI trials.

The most commonly prescribed dose of sole HDR brachytherapy for breast cancer is 34 Gy in ten fractions bid, which is equivalent to a 46 Gy tumor dose using the standard WBI scheme (2 Gy per day, 5 days per week) [20]. Whether the higher local recurrence risk after incomplete tumor excision can be counterbalanced by an additional boost radiotherapy following WBI has not been demonstrated clearly [21]; however, a HDR brachytherapy boost of 13.2 Gy in three fractions following 50 Gy/25 fractions WBI produced favorable local control for close to positive margins [22]. The traditional two X-ray film localization technique used in both this study and other recent reports [8-10] cannot define the actual extent of the target volume and it relates the prescribed dose to the geometry of the implant and not to the target volume. To localize the irregular three-dimensional shape of the target volume and the normal tissue structures correctly and to adapt the reference isodose surface to the shape of this target volume, the utility of brachytherapy planning based on computed tomography imaging has been investigated [23,24]. With sophisticated computed tomography-based implantation and three-dimensional planning system, the target volume definition considering inadequate resection margin foci and the modulation of a higher dose to cover this region might be efficient for those patients who have an insufficient resection margin.

Compared to the postoperative implantation [8-10], intraoperative implantation has the advantages of direct visualization of the excision cavity and shorter local treatment period including surgery and radiotherapy [15]. One disadvantage is the inability to select patients properly for implantation based on definitive pathological findings; however, brachytherapy was used as boost radiotherapy before WBI when the pathology indicated that the patient was unsuitable for brachytherapy alone.

Preliminary guidelines designed to reduce the toxicity of HDR interstitial brachytherapy have been reported [25]. First, ideally, less than 60% of the normal whole breast volume should receive ≥ 50% of the prescribed dose. In this respect, Asian women are at a disadvantage due to their relatively small breasts compared to European and American women, and this might be one of the reasons why APBI has not been actively investigated for them [26]. To satisfy this recommendation, the implementation of computed tomography-based three-dimensional planning would be advantageous. Second, one must minimize hot spots (V_150_) and maintain DHI > 0.75. We found that a higher V_100 _or V_150 _was associated with a significantly higher rate of late subcutaneous toxicity. Mean DHI was low as 0.5, however dose inhomogeneity can make a positive contribution in terms of the tumor control probability [27]. Third, the dose delivered to the skin and chest wall should be less than the prescribed dose. We selected patients with sufficient breast tissue anterior to the tumor and the maximum skin dose was restricted to below 70% of the prescribed dose. Finally, one must proceed with caution if chemotherapy is to be given following APBI. Adriamycin-based chemotherapy after APBI was reportedly related to worse toxicity and cosmesis [25]. We started chemotherapy (mostly not adriamycin-based) during or right after APBI and the chemotherapy use did not affect late toxicity. Previously, we reported the non-inferior safety of concurrent chemoradiotherapy compared to sequential chemoradiotherapy using WBI for early-stage breast cancer [2]. However, the use of larger fraction dose in APBI compared to WBI may necessitate careful administration of chemotherapy. Furthermore, normal tissue changes after APBI have been documented to evolve over time; some endpoint measures (cosmesis, edema, erythema, and breast pain) improved with time, while others (fat necrosis, subcutaneous fibrosis, and telangiectasia) worsened [28,29]. These findings underscore the extended period needed to monitor APBI-related late treatment toxicity, and some patients in this study may need more follow-up to fully evaluate late toxicity.

A few details of the methods need to be mentioned. First, the relatively small breasts in the patients caused concern for treatment toxicity owing to the high irradiated volume/ipsilateral breast volume ratio. Thus, a schedule of 30 Gy in 10 fractions was tried for the first eight patients. After the feasibility and safety of APBI was verified for these eight patients, a dose of 34 Gy in 10 fractions was adopted for the remaining patients. Second, the number of catheters used was small (median 5), with a single plane implant in 17 (35.4%) patients. We tried to remove a tumor and at least 1 cm margin, while at the same time trying to minimize the total excision volume for cosmesis. APBI started shortly (6-9 days) after surgery, and the hemovac drainage was maintained until APBI completed. Accordingly, the excision cavity was less likely to accumulate a hematoma or seroma, and the mean V_100 _was relatively small, at 44.7 cm^3^. A single plane implant was used when the excision cavity was small and flat. However, an increase in catheter number, less use of a single plane implant or computed tomography-based three-dimensional dose planning would be necessary to enhance dose homogeneity.

## Conclusions

In conclusion, APBI using HDR multicatheter interstitial brachytherapy for early-stage breast cancer yielded local control, toxicity, and cosmesis comparable to those of other recent APBI trials or conventional WBI. Our results support the suggestion that APBI is a viable option for select patients with breast cancer. Patients with close resection margins may be ineligible for APBI, and studies of novel brachytherapy techniques should be pursued to optimize APBI outcomes.

## List of abbreviations

BCS: breast-conserving surgery; WBI: whole breast irradiation; APBI: accelerated partial breast irradiation; HDR: high-dose-rate; DHI: dose homogeneity index; V_100 _and V_150_: volumes receiving 100% and 150% of the prescribed dose, respectively; RTOG: radiation therapy oncology group.

## Competing interests

The authors declare that they have no competing interests.

## Authors' contributions

SGY, SH, JK: conception and design, acquisition, analysis and interpretation of data; GHK, JYK, KP: acquisition, analysis and interpretation of data; ESK: analysis and interpretation of data. All the listed authors have been involved in drafting or in revising the manuscript. All authors read and approved the final manuscript.
